# Understanding Depressive Symptoms and Psychosocial Stressors on Twitter: A Corpus-Based Study

**DOI:** 10.2196/jmir.6895

**Published:** 2017-02-28

**Authors:** Danielle Mowery, Hilary Smith, Tyler Cheney, Greg Stoddard, Glen Coppersmith, Craig Bryan, Mike Conway

**Affiliations:** ^1^ Department of Biomedical Informatics University of Utah Salt Lake City, UT United States; ^2^ Department of Psychology University of Utah Salt Lake City, UT United States; ^3^ Department of Family And Preventive Medicine University of Utah Salt Lake City, UT United States; ^4^ Qntfy Crownsville, MD United States; ^5^ Human Language Technology Center of Excellence John Hopkins University Baltimore, MD United States

**Keywords:** social media, Twitter messaging, natural language processing, major depressive disorder, data annotation, machine learning

## Abstract

**Background:**

With a lifetime prevalence of 16.2%, major depressive disorder is the fifth biggest contributor to the disease burden in the United States.

**Objective:**

The aim of this study, building on previous work qualitatively analyzing depression-related Twitter data, was to describe the development of a comprehensive annotation scheme (ie, coding scheme) for manually annotating Twitter data with Diagnostic and Statistical Manual of Mental Disorders, Edition 5 (DSM 5) major depressive symptoms (eg, depressed mood, weight change, psychomotor agitation, or retardation) and Diagnostic and Statistical Manual of Mental Disorders, Edition IV (DSM-IV) psychosocial stressors (eg, educational problems, problems with primary support group, housing problems).

**Methods:**

Using this annotation scheme, we developed an annotated corpus, Depressive Symptom and Psychosocial Stressors Acquired Depression, the SAD corpus, consisting of 9300 tweets randomly sampled from the Twitter application programming interface (API) using depression-related keywords (eg, depressed, gloomy, grief). An analysis of our annotated corpus yielded several key results.

**Results:**

First, 72.09% (6829/9473) of tweets containing relevant keywords were nonindicative of depressive symptoms (eg, “we’re in for a new economic depression”). Second, the most prevalent symptoms in our dataset were depressed mood and fatigue or loss of energy. Third, less than 2% of tweets contained more than one depression related category (eg, diminished ability to think or concentrate, depressed mood). Finally, we found very high positive correlations between some depression-related symptoms in our annotated dataset (eg, fatigue or loss of energy and educational problems; educational problems and diminished ability to think).

**Conclusions:**

We successfully developed an annotation scheme and an annotated corpus, the SAD corpus, consisting of 9300 tweets randomly-selected from the Twitter application programming interface using depression-related keywords. Our analyses suggest that keyword queries alone might not be suitable for public health monitoring because context can change the meaning of keyword in a statement. However, postprocessing approaches could be useful for reducing the noise and improving the signal needed to detect depression symptoms using social media.

## Introduction

### Background

With a lifetime prevalence of 16.2% in the United States [1], major depressive disorder is the fifth biggest contributor to the disease burden in the United States [2]. Several national face-to-face and telephonic interview-based surveys in the United States aim to better understand the prevalence of depressive symptoms in the community. However, these surveys are both episodic and expensive to conduct. Social media platforms like Twitter, in conjunction with “big data” technologies like natural language processing and machine learning, support processing very large datasets and may provide a scalable means of both monitoring depressive disorder over time and providing new insights to better our understanding of depression (and mental illness more generally). As part of our goal of developing language technologies capable of accurately identifying depressive symptoms, we have developed a large manually annotated (coded) corpus or collection of Twitter posts (tweets) coded according to depressive symptoms and psychosocial stressors derived primarily from Diagnostic and Statistical Manual of Mental Disorders, Edition 5 (DSM 5; depressive symptoms) [3] and DSM-IV: Diagnostic and Statistical Manual of Mental Disorders, Edition IV (DSM-IV Axis IV; psychosocial stressors) [4]. This annotated corpus allows us to better understand the language used to express depressive symptoms and psychosocial stressors associated with depression, to identify relationships between depressive symptoms and psychosocial stressors expressed in tweets, and ultimately, to facilitate the development of a natural language processing system capable of automatically identifying depressive symptoms and psychosocial stressors from Twitter data.

#### Social Media

The use of social media for health applications, particularly in the public health domain, is a rapidly growing area of research [5,6]. For example, social media has been leveraged to monitor infectious disease outbreaks [7,8] and understand prescription drug and smoking behaviors [9-11]. The value of social media for understanding mental health is particularly marked, given that it provides—in the case of Twitter—access to public, first person accounts of user behaviors, activities, thoughts, and feelings that may be indicative of emotional well-being [12]. Twitter in particular has several advantages as a resource for data. First, as of August 2015, Twitter has been used by 23% of adults in the United States, with slightly more men (25%) than women (21%) using the service [13]. Second, Twitter is a “broadcast” social network, with all the data public by default. Third, acquiring Twitter data via the free public application programming interface (API) or commercial data resellers (eg, *gnip* [14]) is a relatively straightforward process. However, the use of Twitter data does present a number of challenges. First, the brevity of Twitter posts (≤140 characters) frequently provides insufficient context to confidently interpret a post. Second, the informal nature of the language used in social media posts (eg, “tiredddd”) means that natural language processing techniques and tools developed for more formal texts are likely to perform less well on Twitter data [15]. Third, Twitter posts often exhibit creative spellings and missing spaces (eg, “sodepressed”), presenting challenges for automatic processing. Finally, Twitter users may selectively discuss topics of interest with researchers; for example, some individuals may not feel comfortable discussing disease-related symptoms on social media due to concerns of privacy and stigmatization [16].

#### Major Depressive Disorder

The American Psychiatric Association defines major depressive disorder as continuously experiencing *depressed mood* and *anhedonia* for 2 weeks or more as well as one or more of the following symptoms: *fatigue*, *inappropriate guilt*, *difficulty concentrating*, *psychomotor agitation or retardation*, or *weight loss or gain* [3,4]. These symptoms make major depressive disorder one of the most debilitating and burdensome global diseases [17,18], with an economic impact estimated to be US $2.5 trillion in 2010 [19]. For individuals living with depression, the disorder can substantially reduce quality of life in several areas, including interactions with others, productivity at work, and quality of sleep and nutrition [20]. Depression has also been correlated with other high-risk behaviors and chronic diseases, including smoking [21], alcohol consumption [22], physical inactivity [23], and sleep disturbance [20,24].

#### Population-Level Depression Surveys

Given the range and extent that depression affects a given population, several surveys, programs, and diagnostic tools have been developed to better understand or diagnose depressive disorder. For example, in the United States, the *National Survey on Drug Use and Health (NSDUH)* provides national, state, and local data related to alcohol, tobacco, illegal drug use and abuse, and mental disorders, including nonincarcerated citizens of age 12 and older [25]. The *Youth Risk Behavior Surveillance System (YRBSS)* monitors behaviors such as alcohol and other drug use, tobacco use, and unhealthy dietary behaviors, and so on, and their correspondence with death and disability among youth and adults [26]. *The Behavioral Risk Factors Surveillance System (BRFSS)* is a telephone survey that collects data from across the United States, including health-related risk behaviors, chronic health conditions, and use of preventive services [27]. The *BRFSS - Anxiety and Depression Optional Module* specifically collects information at the state level to assess the prevalence of anxiety and depressive disorders with questions that closely mirror the DSM 5 major depression criteria.

### Related Works

#### Major Depressive Disorder and Social Media

Recent work at the intersection of computer science, public health, and psychology suggests that social media can be leveraged to better understand, identify, and characterize depression [12]. For example, De Choudhury et al used a crowdsourcing data generation method in conjunction with machine learning to identify depression-indicative tweets at scale [28], whilst a follow-up study investigated the characteristics of Twitter users prior to the onset of depression, discovering that *decrease in social activity*, *raised negative affect*, *highly clustered ego networks*, *heightened relational and medical concerns*, and *greater expression of religious involvement* were all characteristic of the onset of depression [29].

In a study using Facebook, Schwartz et al used status updates and personality survey results as features in a regression model to classify the degree of depression of 28,749 Facebook users [30]. A temporal analysis of these posts demonstrated that mood worsens in the transition from summer to winter for users. Coppersmith et al further characterized the language of mental illnesses (eg, depression) by identifying tweets containing self-reported diagnosis (“I was diagnosed with depression today”), then analyzing the linguistic characteristics of tweets from both a self-reported depression and a control group, observing that the usage of words from the Linguistic Inquiry and Word Count (LIWC) lexicon [31] associated with negative emotions including *anxiety* and *anger*, biological states such as *health* and *death*, cognitive mechanisms including *cause* and *tentativeness*, and syntactic usage of *first person pronoun* (eg, “I”) may distinguish a depressed from a nondepressed individual [32,33]. Preotuic-Pietro et al observed many features that distinguish depressed Twitter users from controls [34], for example, terms associated with *illness management* (eg, “meds,” “pills,” and “therapy”) and *increased focus on the self* (eg, “I,” “I am,” “I have,” “I was,” and “myself”).

In this study, we build on these existing efforts by developing an annotation scheme for encoding depressive symptoms and psychosocial stressors associated with major depressive disorder in Twitter tweets and conducting analyses to provide insights into how users express these symptoms on Twitter. From these analyses, specifically, we aim to (1) validate the annotation scheme, (2) learn the predictive value of depression-related keywords with respect to identifying depressive symptoms and psychosocial stressors, (3) determine the frequency of depressive symptoms and psychosocial stressors expressed, (4) learn new predictive words for each depressive symptom and psychosocial stressor, and (5) assess whether particular depressive symptoms and psychosocial stressors are correlated with one another.

## Methods

### Developing a Depression Annotation Scheme and Corpus for Twitter

All the data were collected from the Twitter API complying with Twitter’s terms of service.

#### Developing an Annotation Scheme

In order to understand the various ways indicators of major depression disorder could be expressed in tweets and address our goal of building a dataset that can be used to train and test machine learning algorithms, we developed an annotation scheme (coding scheme) based on 6 resources:

Depression symptoms as described in the *Diagnostic and Statistical Manual of Mental Disorders, Edition 5 (DSM-V) [3]*;

Psychosocial stressors described in *Axis IV of the Diagnostic and Statistical Manual of Mental Disorders, Edition IV (DSM-IV) [4]*;

Depression symptoms described in the *Behavioral Risk Factors Surveillance System—Depression Module [27]*;

Depression symptoms described in the *Harvard Department of Psychiatry National Depression Screening Day Scale (HANDS) [35]*;

Depression symptoms described in the *Patient Health Questionnaire (PHQ-9) [36]*;

Depression symptoms described in the *Quick Inventory of Depressive Symptomatology (QIDS-SR) [37]*; and

Suicide risk factors derived from the *Columbia Suicide Severity Scale [38]* **.**

**Figure 1:**
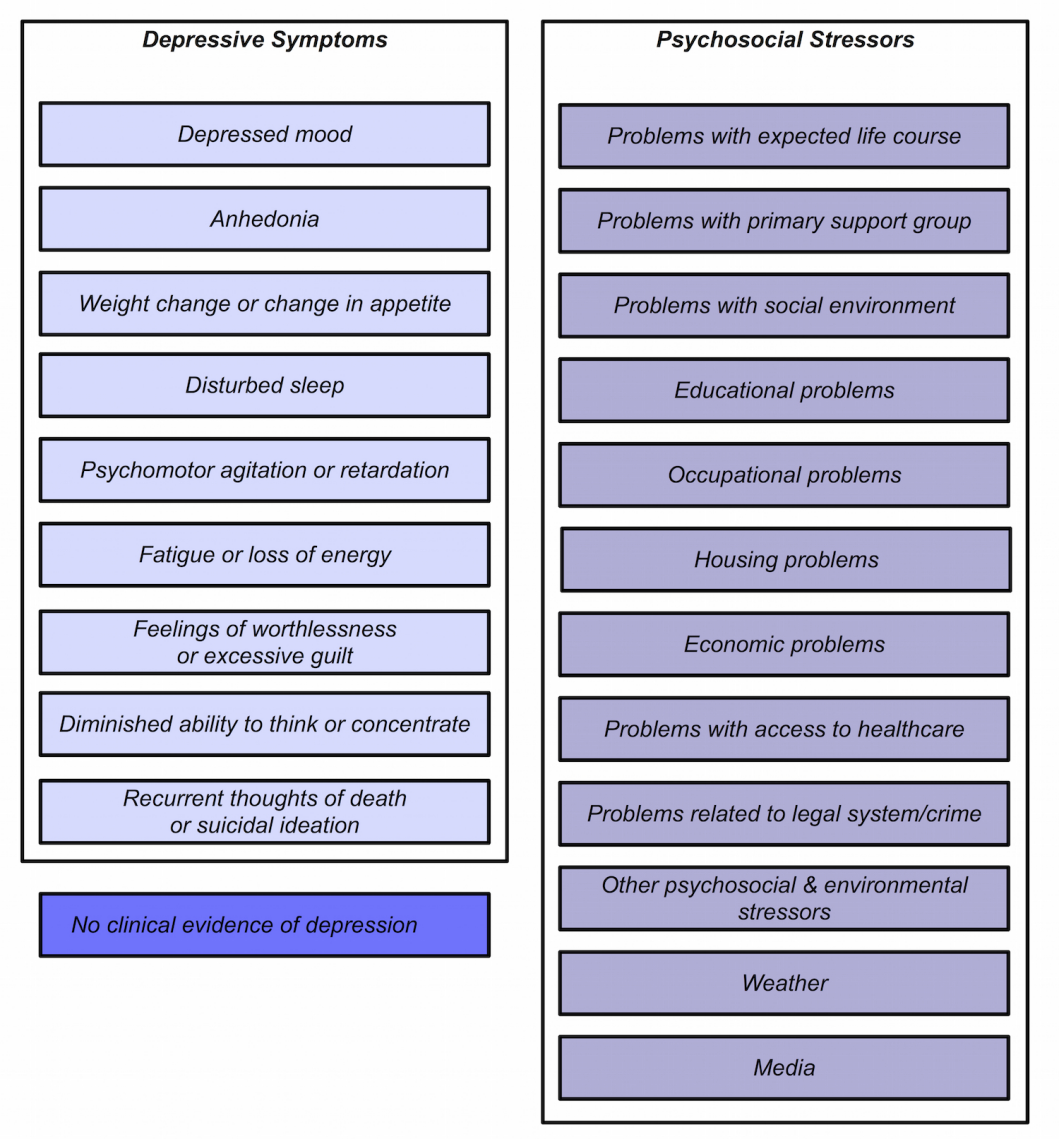
Major depressive disorder scheme (parent categories).

Finally, we enriched the annotation scheme with additional depression-related categories observed frequently in the data (*weather* and *media*). The resulting scheme contains depression symptom categories (9 parent categories) and psychosocial stressor categories (12 parent categories; Figure 1) [39]. Before finalizing the annotation scheme, both a psychiatrist and a counseling psychologist provided feedback on the annotation categories chosen and annotation instructions.

#### Building a Depression-Related Twitter Corpus

Data for our depression-related Twitter corpus were collected in two distinct ways. First, for our primary corpus construction effort, we searched the Twitter API using depression-related terms (Depressive Symptom and Psychosocial Stressors Acquired Depression, *SAD,* corpus). Second, we sampled the data collected as part of the *2015 Computational Linguistics and Clinical Psychology* (CLPsych) Shared Task [40]. Both corpora are described in detail below.

##### Depressive Symptoms and Psychosocial Stressor Acquired Depression (SAD) Corpus

We randomly selected Twitter user tweets from March 1 to March 31, 2013, using the Twitter API. For each day in March 2013, we randomly selected 300 tweets containing one or more keywords from the LIWC lexicon (eg, “die,” “pain,” and “tired”). We used the LIWC “sad” category keyword list and augmented this list with several keywords selected by a board-certified clinical psychologist (author CB). For example, the presence of the keyword “insomnia” might be suggestive of the depression symptom *disturbed sleep*. A complete list of keywords and associated depression stressors and symptoms can be found in Table 1 (n=110 total keywords).

**Table 1:** Linguistic Inquiry and Word Count (LIWC) concepts and associated keywords^a^.

Depression categories		Linguistic Inquiry and Word Count
**Depressive symptoms**		
	Depressed mood	pain
	Weight change or change in appetite	appetite
	Disturbed sleep	insomnia
	Psychomotor agitation or retardation	restless, jitter*, groggy, dazed
	Fatigue or loss of energy	tired
	Feelings of worthlessness or excessive inappropriate guilt	guilt*, burden
	Diminished ability to think or concentrate, indecisiveness	concentrat*, focus*, indeci*
	Recurrent thoughts of death, suicidal ideation	suicid*, kill
**Psychosocial stressors**		
	Problems with primary support group	death, die*, funeral, cremat*,
		divorc, abus*, neglect*
	Occupational problems	fired, unemploy*
	Housing problems	homeless*
	LIWC “sad” keyword	abandon*, ache*, aching, agoni*,
		alone, broke*, cried, cries, crushed,
		cry, damag*, defeat*, depress*,
		depriv*, despair*, devastat*,
		disadvantage*, disappoint*,
		discourag*, dishearten*, disillusion*,
		dissatisf*, doom*, dull*,
		empt*, fail*, fatigu*, flunk*,
		gloom*, grave*, grief, griev*,
		grim*, heartbr*, helpless*, homesick*,
		hopeless*, hurt*, inadequa*, inferior*,
		isolat*, lame*, lone*, longing*,
		lose, loser*, loses, losing,
		loss*, lost, melanchol*, miser*,
		mourn*, neglect*, overwhelm*
		pathetic*, pessimis*, piti*, pity* ,
		regret*, reject*, remorse*, resign*,
		ruin*, sad, sobbed, sobbing, sobs,
		solemn*, sorrow*, suffer*, tears*,
		traged*, tragic* , unhapp*,
		unimportant, unsuccessful*, useless*,
		weep*, wept, whine*, whining,
		woe*, worthless*, yearn*

^a^Depressive symptom *anhedonia* and psychosocial stressors such as *problems with expected life course with respect to self, problems related to the social environment, educational problems, economic problems, problems with access to health care, problems related to the legal system and crime, other psychosocial and environmental problems, weather,* and *media* do not have associated keywords.

##### CLPsych Corpus

In addition to the SAD corpus, we sampled tweets from a large corpus of Twitter data developed for the *2015* CLPsych shared task [40]. In order to build this corpus, CLPsych researchers queried Twitter (via the public Twitter API) for users with a self-disclosed, publicly stated psychiatric diagnosis (eg, “I was diagnosed with having depression”), then collected all available tweets from that user. The corpus consisted of up to 3000 tweets from each of the 477 users, from which we randomly sampled 100 users with self-disclosed depression diagnosis from the CLPsych dataset, located the “self-diagnosis” tweet, then annotated the subsequent 10 tweets from that user using our annotation scheme.

### Validating the Annotation Scheme

In order to validate our annotation scheme, 3 annotators—2 psychology graduate researchers and a postdoctoral biomedical informatics researcher—annotated 1200 tweets from the SAD corpus in 3 phases. In phase 1, all 3 annotators annotated 300 tweets and reached agreement with consensus review. In phase 2, for the remaining 900 tweets and for all annotator pair combinations, 2 annotators independently annotated 300 tweets, and the remaining third annotator adjudicated any disagreements. For example, if annotators A1 and A2 annotated 300 tweets, annotator A3 would adjudicate those tweets where A1 and A2 disagreed regarding the appropriate label. We compared the annotations between each pair of annotators to determine the asserted categorical matches and mismatches. For example, a match occurs when both annotators (eg, A1 and A2) annotated the same category for the same tweet. There are 2 types of mismatches: type 1 mismatch occurs if A1 annotated a category for a tweet not annotated by A2; and a type 2 mismatch if A2 annotated a category for a tweet not annotated by A1. We report both overall and granular inter-annotator agreement between annotator pairs by comparing one annotator’s annotations to the other’s annotations (rather than assuming a ground truth) to compute *F* score [41]. *F* score is computed from the matches and mismatches and given as a percentage from the following equation:

*F* score=(2×matches)/([2×matches]+mismatches]) × 100%

**Figure 2:**
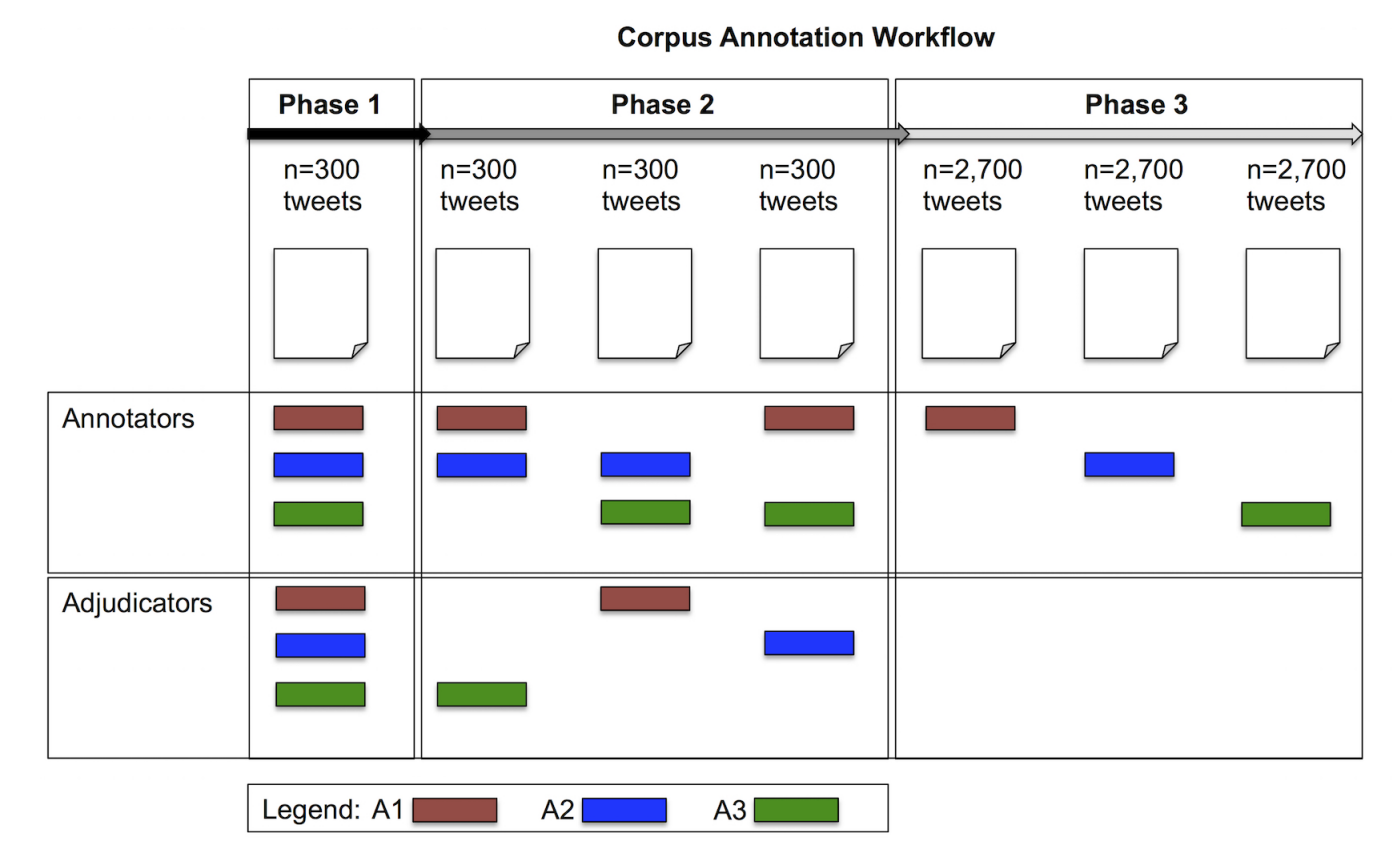
SAD corpus annotation in phases. A#=Annotator eg, A1=Annotator 1. SAD: Depressive Symptom and Psychosocial Stressors Acquired Depression.

In phase 3, each annotator independently annotated 2700 tweets (8100 tweets total from 3 annotators) and to further ensure reliability, 1200 tweets were annotated by all 3 annotators. The resulting SAD corpus consists of 9300 tweets. A summary of this annotation workflow can be found in Figure 2. The CLPsych corpus was annotated by 1 annotator resulting in 1019 tweets (which are not included in the 9300 SAD tweets).

### Learning the Predictive Value of Depression-Related Keywords

For both the SAD and CLPsych corpora, in order to assess how accurately these depression-related keywords could identify depression-related tweets, we computed the precision of each depression-related keyword, defined as the count of tweets identified by the depression-related keyword and associated with a depression-related category divided by the total count of tweets identified by the depression-related keyword (tweet hits). For example, if 4 tweets were identified by the keyword “sobbing,” but only 1 of the 4 total tweets was encoded as a depressive symptom or psychosocial stressor, then the precision of the depression-related keyword is 25%. We classified the resulting precision using 5 equally sized categories:

1. zero to poor precision: 0-19%,

2. poor to low precision: 20-39%,

3. low to moderate precision: 40-59%,

4. moderate to high precision: 60-79%, and

5. high to excellent precision: 80-100%.

For each corpus and each precision category, we report the count of tweets identified by the count of depression-related keywords (tweet hits). Specifically, one or more keywords can match a single tweet, for example, the keywords “depressed” and “fired” in “I’m so depressed because I got fired today”; therefore, our denominator is the number of times a keyword was matched in a tweet.

### Exploring the Frequency of Symptoms and Psychosocial Stressors

In order to estimate the proportion of said depressive symptoms and psychosocial stressors in our corpus, we characterized our total corpus of tweets by the proportion of tweets representing *no evidence of clinical depression* and *evidence of clinical depression*. Of the tweets representing evidence of clinical depression, we report the proportion of tweets representing depressive symptoms and psychosocial stressors. Finally, we provide example subtypes of depressive symptoms and psychosocial stressors. We compared the distributions of annotation categories between the SAD and CLPsych corpora in order to identify salient characteristics of Twitter users with a publicly stated diagnosis of depression.

### Determining Predictive Word Features for Depressive Symptoms and Psychosocial Stressors

For both the SAD and CLPsych corpora, in order to identify words and phrases most characteristic of each category of depressive symptoms and psychosocial stressors (eg, the words most characteristic of, say, *occupational problem*), we used a technique referred to as feature selection [42] (keyword extraction in the corpus linguistics literature [43]). More specifically, we used the information gain metric [44] to compare the relative frequency of words associated with each depression category (eg, the word “fired” may appear more frequently in the *occupational problem* category than the *educational problem* category). The 10 most characteristic words—identified by information gain—are reported for each category. Specifically, we used Weka version 3.16.13 to learn words that occurred with the highest average rank for 5 independent subsets of the dataset [42].

### Assessing Correlations Between Depressive Symptoms and Psychosocial Stressors

For the 9300 tweet SAD corpus only, in order to determine whether a correlation exists between 2 specific depressive symptoms and psychosocial stressors, we computed Pearson correlation coefficients for each pairwise combination of the 21 parent categories of depressive symptoms and psychosocial stressors from the annotation scheme. Given that each symptom or stressor category has only 2 states (annotated or not annotated), this correlation coefficient is sometimes called the phi coefficient, although the phi and Pearson correlation coefficients are algebraically identical. A higher correlation coefficient indicates that when the psychosocial stressor is annotated, the depressive symptom is more likely to also be annotated. We used the *r* value to interpret magnitude because *P* values are affected by sample size, whereas *r* values are not. We classified the correlation magnitude using Cohen effect size criteria into 4 categories [45]: less than small effect: <0.09; small to medium effect: 0.1-0.29; medium to large effect: 0.3-0.49; and greater than large effect: >0.50.

## Results

### Characterizing the Corpus

Our depression disorder scheme is comprised of 9 depressive symptoms and 12 psychosocial stressor categories that were applied to the SAD and CLPsych Twitter corpora. We observed an average number of 14-15 words with a standard deviation between 7 and 8 words (Table 2).

**Table 2:** Comparison of characteristics by corpus.

Characteristic	SAD	CLPsych
Query-level	tweet-level	user-level
Number of unique tweets	9300	1019
Number of unique words	19,822	3258
Average number of words per tweet (SD)	14.56 (7.40)	15.44 (8.07)

### Validating the Annotation Scheme

We observed high overall interannotator agreement (*F* scores) between annotator pairs: ranging from 76% to 81% (Table 3). Overall *F* scores dropped slightly when comparing matches for all 3 annotators. Across pairs, we observed *F* scores ranging from 86% to 89% for *no evidence of clinical depression*. *F* scores varied widely across all annotated categories. High *F* scores were observed across annotator pairs for the depression symptom *fatigue or loss of energy* and psychosocial stressors *recurrent thoughts of death and suicidal ideation*.

**Table 3:** For the SAD corpus, interannotator agreement (*F* scores) between annotators according to depressive symptoms and psychosocial stressors. — means category not observed by annotators.

Depression categories	A1/A2, (%)	A2/A3, (%)	A1/A3, (%)
Overall	81	78	76
No evidence of depression	89	86	87
Symptoms			
Depressed mood	38	60	48
Anhedonia	–	–	–
Weight change or change in appetite	–	0	100
Disturbed sleep	100	50	0
Psychomotor agitation or retardation	–	–	–
Fatigue or loss of energy	74	78	94
Feelings of worthlessness or excessive inappropriate guilt	0	29	68
Diminished ability to think or concentrate, indecisiveness	100	–	0
Recurrent thoughts of death, suicidal ideation	100	100	75
Stressors			
Problems with expected life course with respect to self	0	0	0
Problems with primary support group	0	40	36
Problems related to the social environment	23	42	58
Educational problems	–	50	0
Occupational problems	–	0	–
Housing problems	–	0	–
Economic problems	–	67	50
Problems with access to health care	–	0	–
Problems related to the legal system and crime	–	0	0
Other psychosocial and environmental problems	–	0	0
Weather	–	100	–
Media	50	0	67

### Learning the Predictive Value of Depression-Related Keywords

**Figure 3:**
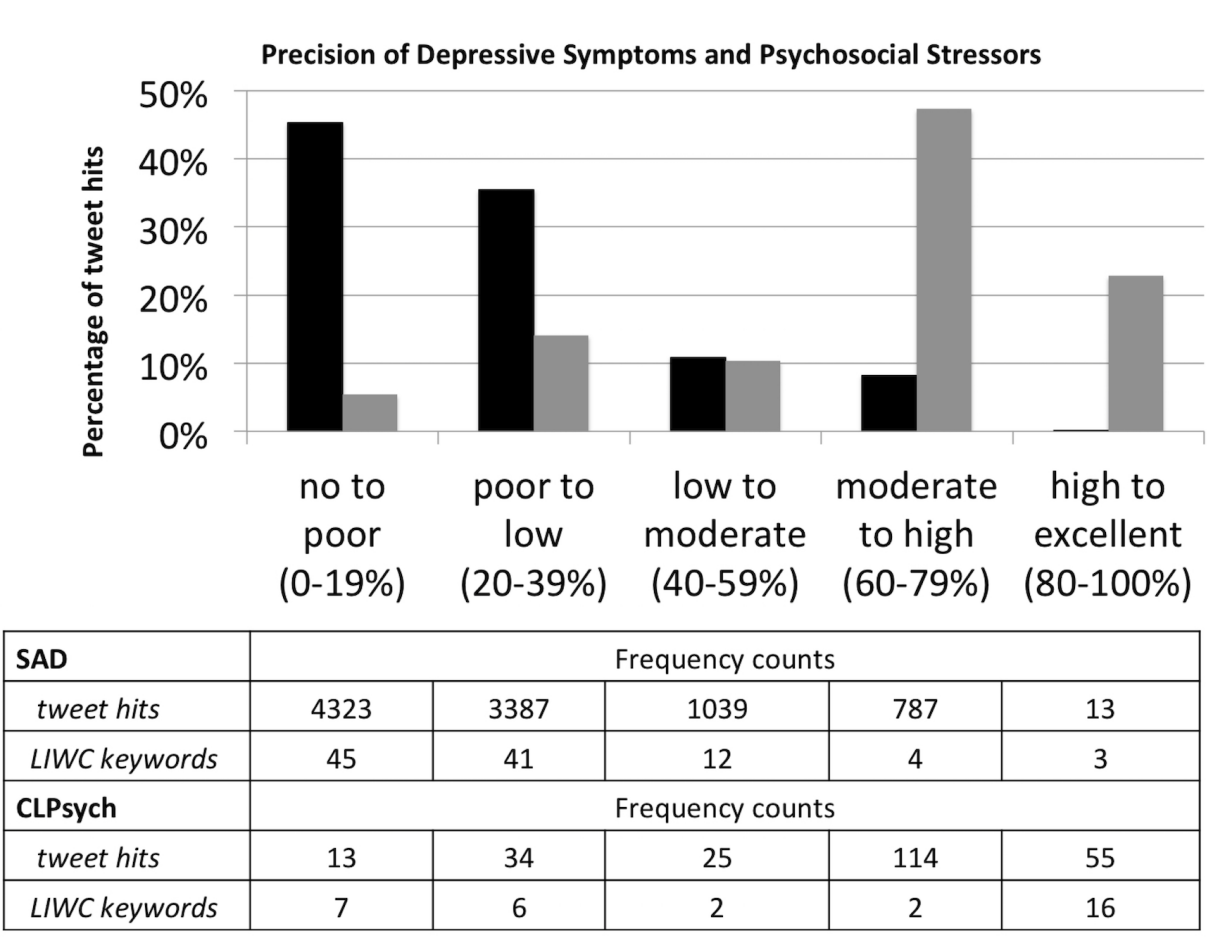
Distribution of tweet hits by precision with LIWC Keyword counts for each corpus. Black bars=SAD corpus; Gray bars= CLPsych corpus. SAD: Depressive Symptom and Psychosocial Stressors Acquired Depression.

For the SAD corpus, of the 110 unique depression-related keywords, 105 keywords were found corresponding to 9549 nonmutually exclusive tweet hits. We observed a range of precision across depression-related keyword hits: 45.27% (4323/9549) zero to poor, 35.47% (3387/9549) poor to low, 10.88% (1039/9549) low to moderate, 8.24% (787/9549) moderate to high, and 0.14% (13/9549) high to excellent (Figure 3). For the CLPsych corpus, the 35 unique depression-related keywords found correspond to 241 nonmutually exclusive tweet hits. We observed a range of precision across depression-related keyword hits: 5.40% (13/241) zero to poor, 14.11% (34/241) poor to low, 10.37% (25/241) low to moderate, 47.30% (114/241) moderate to high, and 22.82% (55/241) high to excellent.

### Exploring the Frequency of Depressive Symptoms and Psychosocial Stressors

**Figure 4:**
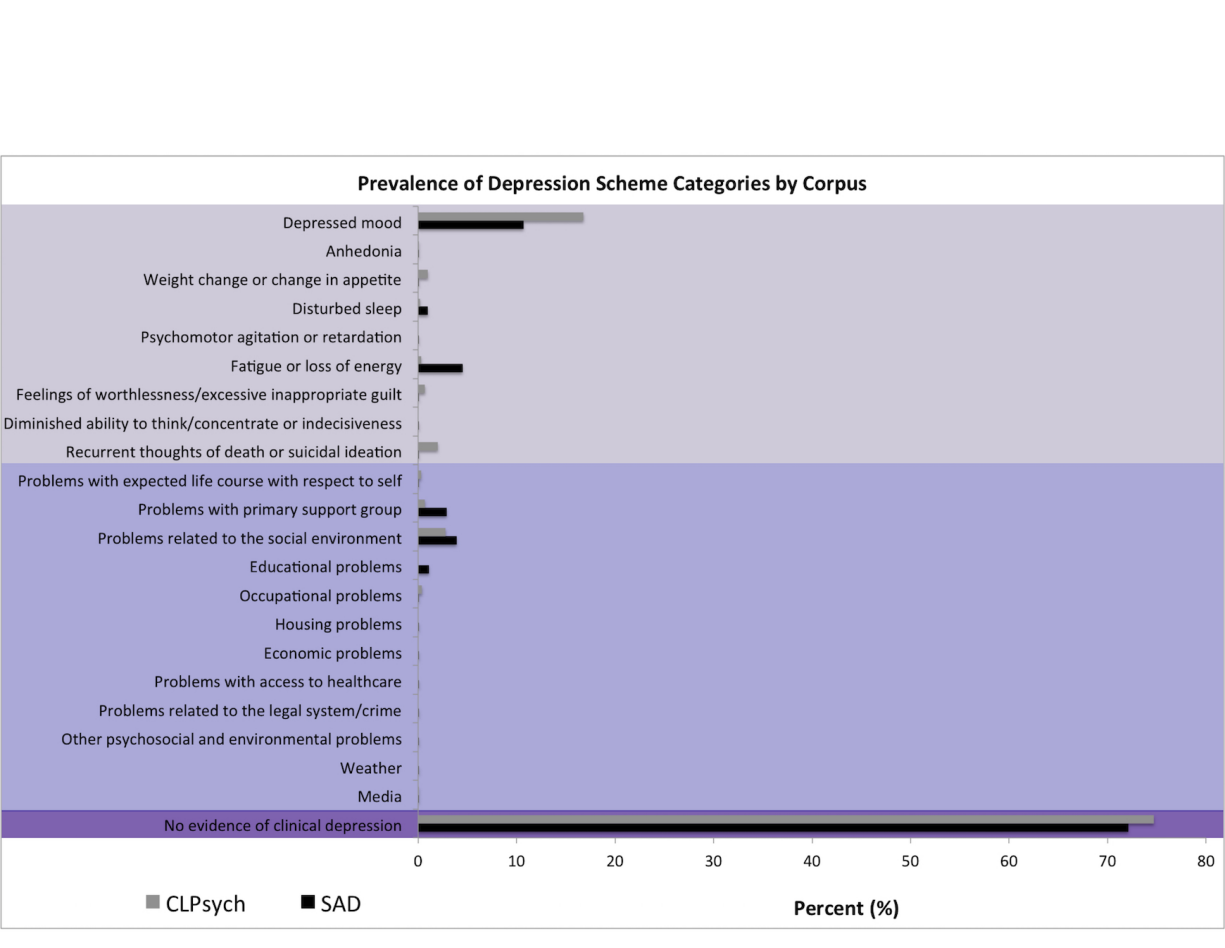
Prevalence of categories by corpus. Light purple: depressive symptoms, medium purple: psychosocial stressors, dark purple: no evidence of clinical depression.

The SAD corpus consists of 9300 tweets. Of these tweets, 9293 were annotated with one or more categories from our scheme: 1 category (98.11%, 9117/9293), 2 categories (1.86%, 173/9293), and 3 or more categories (<1%, 3/9293). Overall, we observed a total of 9473 category annotations with the following distribution of categories annotated per tweets. A total of 72.09% (6829/9473) of annotations represent *no evidence of clinical depression* (Figure 4). Of the 27.91% (2644/9473) annotations that contained *evidence of clinical depression*, 18.20% (1724/9473) represented depressive symptoms and 9.71% (920/9473) represented psychosocial stressors. The CLPsych corpus consists of 1019 tweets. All tweets were annotated with only 1 category from our scheme. About 74.68% (761/1019) of annotations represent *no evidence of clinical depression*. Of the 25.32% (258/1019) annotations that contained *evidence of clinical depression*, 19.04% (194/1019) represented depressive symptoms and 6.28% (64/1019) represented psychosocial stressors.

### Determining Predictive Word Features for Depressive Symptoms and Psychosocial Stressors

**Figure 5:**
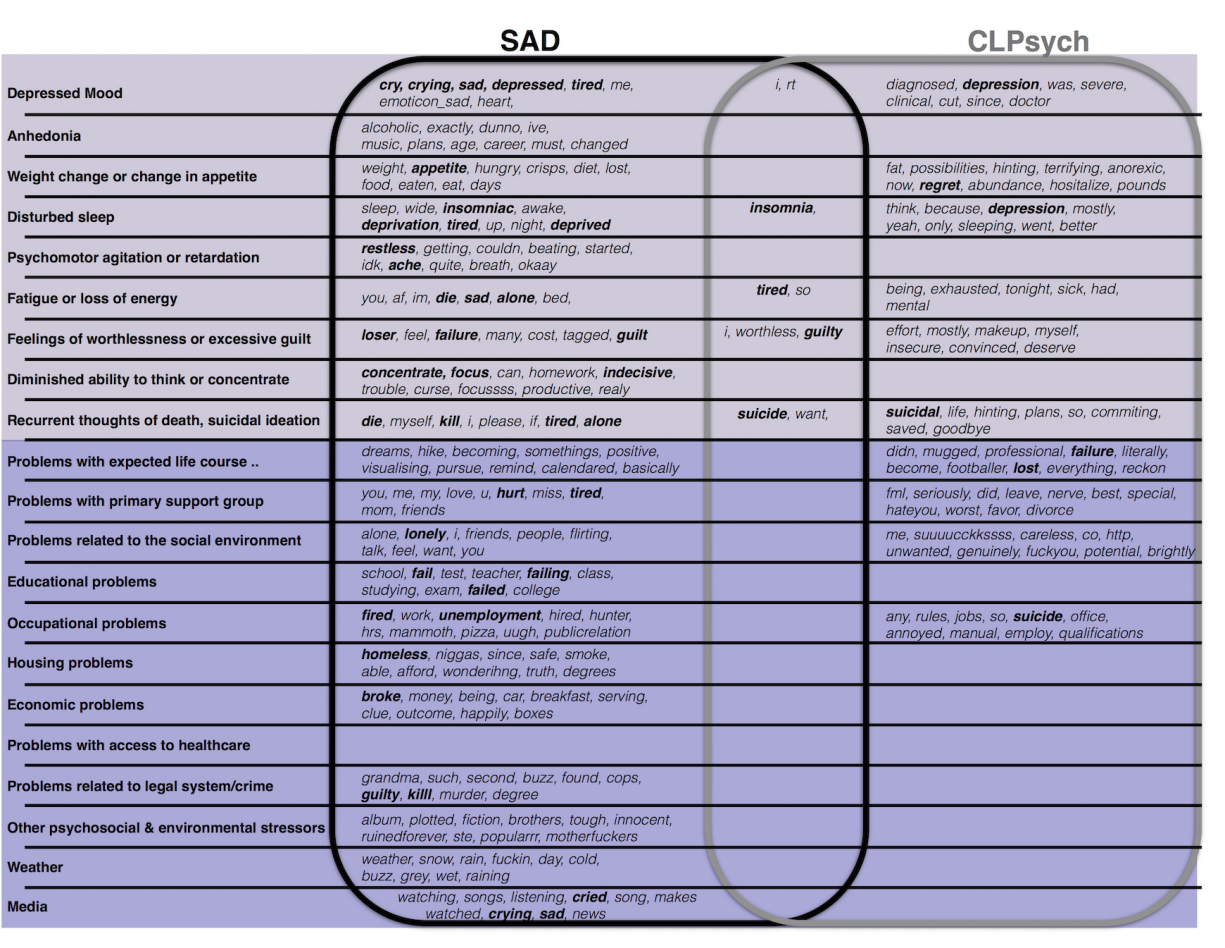
Most informative terms classified with associated depressive symptoms and psychosocial stressors. Shared terms occur at the intersect of the circled lists.

For the SAD corpus, 31 words were identified as the most informative features for classifying tweets for 11 depressive symptoms and psychosocial stressor categories (Figure 5). About 19 of these terms are also covered by the original LIWC keyword list.

### Assessing Correlations Between Depressive Symptoms and Psychosocial Stressors

**Figure 6:**
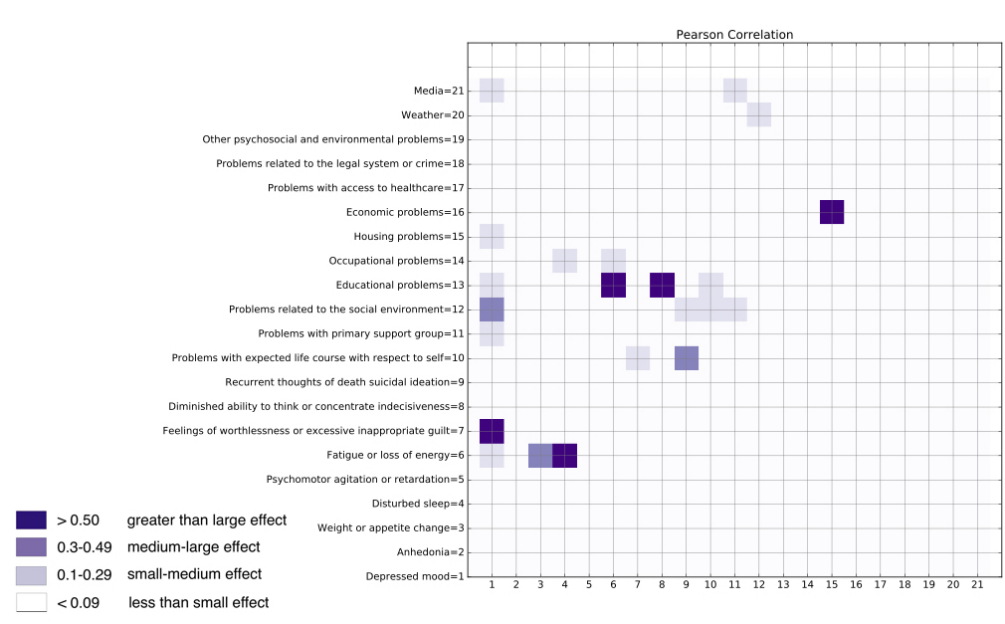
SAD heat map of tweet-level, depressive symptom, and psychosocial stressor cooccurrences. Darker means larger measure of Cohen effect size; lighter means smaller measure of Cohen effect size. The number that indexes the category on the y-axis also corresponds to the category for the x-axis. For example, if “Depressed mood=1” appears on the y-axis, then “1” on the x-axis corresponds to the category “Depressed mood.” SAD: Depressive Symptom and Psychosocial Stressors Acquired Depression.

In terms of depressive symptoms and psychosocial stressors, we observed 5 pairs with higher than large correlations, 3 pairs with medium to large correlations, and 13 with small correlations (Figure 6). Furthermore, all other possible combinations were either of low effect (≤0.09) or not observed in the corpus. Specifically, *fatigue or loss of energy* demonstrated large effect with *disturbed sleep* and *educational problems*. *Depressed mood* had large effect with *feelings of worthlessness* or *excessive inappropriate guilt*. *Educational problems* had large effect with *fatigue or loss of energy* and *diminished ability to think or concentrate* and *indecisiveness*. *Housing problems* and *economic problems* also demonstrated a large effect.

## Discussion

### Principal Findings

In summary, several depressive symptoms and psychosocial stressor categories could be observed in the corpus. For tweets containing two or more categories, we found large correlations between some depressive symptoms and psychosocial stressor categories. Our assessment also suggests that keyword queries alone might not be suitable for public health monitoring.

### Characterizing the Corpus

We conducted an annotation study to investigate methods for effective data collection and understand how people tweet about depression on Twitter. We observed similar average number of tokens and standard deviations for both the SAD and CLPsych corpora (Table 2).

### Validating the Annotation Scheme

In order to address these aims, we applied our scheme to the SAD corpus. We observed that annotators are able to discern tweets representing *no evidence of clinical depression* and achieve high overall *F* scores (acceptable within the NLP community [46]; Table 3). However, we observed variable *F* scores for depressive symptoms and psychosocial stressors, which we attribute to the lower prevalence of these categories in the corpus.

### Learning the Predictive Value of Depression-Related Keywords

Specifically, we assessed the predictive value of depression-related keywords for effective data collection because the mechanism for collecting data, the Twitter API, can only apply keywords to retrieve relevant tweets. We observed different distributions of precision between the SAD and CLPsych corpora (Figure 3). For the SAD corpus, most depression-related keywords demonstrated zero to poor to low precision. In contrast, the CLPsych corpus, most depression-related keywords demonstrated moderate to high to excellent precision. We hypothesize that the depression-related keywords have better precision because of the lack of ambiguity in their usage due to contextual grounding with the self-reported diagnosis (“I was diagnosed with depression”). Specifically, for the SAD corpus, less than 1% of the tweets were classified as high to excellent precision that were identified by querying tweets with 3 depression-related keywords: “inferior,” “dishearten,” and “restless.” For example, “Everyday leaves me feeling more hopeless and restless.” In contrast, for the CLPsych corpus, more than 22% of the tweets were classified with high to excellent precision which were identified by querying tweets with 15 depression-related keywords such as “inferior,” “dishearten,” “depressants,” “suicidal,” “tired,” “miserable,” “depressive,” “suicide,” “divorce,” “unhappy,” “heartbreak,” “lonely,” “insomnia,” “depressing,” and “hurts.” For example, “I always feel insecure and inferior to everyone in my life.” From this assessment, we will leverage these depression-related keywords to query tweets related to depressive symptoms: *depressed mood*, *disturbed sleep*, *fatigue or loss of energy*, *feelings of worthlessness or excessive inappropriate guilt*, as well as psychosocial stressors: *recurrent thoughts of death, suicidal ideation*, *problems with primary support group*, and *problems related to the social environment*.

### Exploring the Frequency of Symptoms and Psychosocial Stressors

Overall, we observed similar distributions of *no evidence of clinical depression* and *evidence of clinical depression* categories as well as depressive symptoms and psychosocial stressors between the SAD and CLPsych Corpora (Figure 4). We observed a skewed distribution of depressive symptoms and psychosocial stressors categories in both corpora. The most prevalent category for both corpora was *no evidence of clinical depression* meaning for every 10 tweets reviewed 7 were not relevant. This finding suggests that our a priori depression-related keyword lexicon was insufficient for consistently identifying depression-related tweets and that natural language processing methods will be required to increase accuracy.

When evidence of clinical depression was identified for both the SAD and CLPsych corpora, tweets more often described depressive symptoms rather than psychosocial stressors. This finding suggests that Twitter users may be more comfortable or feel an immediate need to describe their current mental state and physical feelings (eg, “I can’t concentrate”) rather than the psychosocial stressors that may have given rise to these depressive symptoms (eg, “I can’t concentrate because of my recent car accident”). In terms of depressive symptoms, both corpora contained *depressed mood* as the most prevalent depressive symptom. However, for the SAD corpus, the following second and third most prevalent depressive symptoms included *fatigue or loss of energy* and *disturbed sleep*; in contrast to the CLPsych corpus, in which the following second and third most prevalent depressive symptoms included *weight change or change in appetite* and *feelings of worthlessness or excessive inappropriate guilt*. In terms of psychosocial stressors, both corpora contained *problems related to the social environment* and *problems with primary support group*. However, for the SAD corpus, the third most prevalent psychosocial stressor included *educational problems*; whereas, for the CLPsych corpus, the third most prevalent psychosocial stressors included *recurrent thoughts of death and suicidal ideation*. The SAD depressive symptoms and psychosocial stressor distributions are unsurprising and mirror the distributions found in our pilot annotation effort [39,47].

### Determining Predictive Word Features for Depressive Symptoms and Psychosocial Stressors

To expand on our data acquisition approach and supplement the depression-related keyword lexicon, we also conducted a feature selection study to identify words most characteristic of each depression symptom and psychosocial stressor with the aim of identifying new keywords not already present in our lexicon of depression-related keywords. For the SAD corpus, only one category— *problems with access to health care* —had too few mentions to learn new keywords (Figure 5). Of the most informative keywords identified, most were absent from our handcrafted depression-keyword lexicon, suggesting that some new words could be useful for pulling relevant tweets for most depressive symptoms and psychosocial stressor categories. For the CLPsych corpus, we observed many new informative words. However, only about half of the categories had more than 2 mentions. Few depression-related words were shared between the SAD and CLPsych corpora, suggesting that we may still learn new words. Similar to Coppersmith et al [32] and Preotuic-Pietro et al [34], our work indicates that greater use of personal pronouns could indicate an increased focus on the self. We also observed words for many depressive symptoms and psychosocial stressors associated with *anxiety* and *anger* and biological states such as *health* and *death*. These new words are promising; however, we leave it to future studies to determine their precision or recall on a new, unseen Twitter dataset.

### Assessing Correlations Between Depressive Symptoms and Psychosocial Stressors

In terms of depressive symptoms and psychosocial stressors, we observed 5 pairs with higher than large effects (Figure 6). Specifically, *fatigue or loss of energy* demonstrated large effects with another depressive symptom of *disturbed sleep* and psychosocial stressor of *educational problems*. Our analysis suggests that individuals expressing chronic fatigue describe this symptom affecting their quality of life including difficulties in managing sleep and nutrition, productivity at work or school, and interactions with others [20]. *Depressed mood* demonstrated large effect with another depressive symptom of *feelings of worthlessness or excessive inappropriate guilt*. Other interesting and intuitive findings are that *educational problems* exhibited large effects with other symptoms of *fatigue or loss of energy* and *diminished ability to think or concentrate and indecisiveness*, suggesting that if an individual experiences problems during his or her academic studies it could be attributed to tiredness and the inability to concentrate on subject matter. *Housing problems* and *economic problems* also demonstrated large effect, a fact that makes sense intuitively if we consider that an individual experiencing economic problems may encounter difficulties maintaining a home.

### Limitations

For the SAD corpus, we cannot confirm whether an individual Twitter user has or has not received a formal diagnosis of depression. However, many individuals go undiagnosed for depression; therefore, one advantage of this methodology is that it could capture relevant symptomology without a formal diagnosis. However, it is important to be clear that for ethical reasons (eg, individual privacy) the intent of this tool is not to diagnose depression or attempt to intervene at the individual level, but rather to estimate and report the prevalence of depression symptoms at the population level over time in the United States. Furthermore, the correlational analysis performed on the SAD corpus could not be performed for the smaller CLPsych corpus, as we did not observe more than one depression symptom or psychosocial stressor associated with each tweet.

### Comparison With Prior Work

Since our pilot study on a dataset of 500 depression-related tweets [39,47], little research has been conducted specifically to qualitatively (rather than computationally) understand the range of depression-related symptoms that manifested in Twitter data. An important exception is Cavazos-Rehg et al, who used a qualitative technique to study 2000 randomly selected tweets containing one or more depression-related keywords (depressed, #depressed, depression, #depression), finding that two-thirds of the tweets either described depressive symptoms, or expressed thoughts consistent with depression [48]. This study complements and builds on that reported in Cavazos-Rehg et al in several key ways. First, the primary dataset leveraged in this study is almost 5 times larger than that used by Cavazos-Rehg et al (9300 tweets and 2000 tweets, respectively). Second, the dataset used in this study was created using a variety of keywords related to depression and depressive symptoms (110 in total) rather than Cavazos-Rehg et al’s use of lexical variants of the word “depression.” Third, this study extends beyond the analysis of DSM 5 depressive symptoms to include psychosocial stressors derived from DSM-IV Axis IV [4] (eg, *educational problems*, *occupational problems*, *problems related to the social environment*). Finally, this study is designed to investigate correlations between depression symptoms and psychosocial stressors.

This study has 2 main goals: First, to provide insights into how users express depressive symptoms on Twitter; and second, to create a dataset (ie, an annotated corpus of depression-related tweets) suitable for both training and testing natural language processing algorithms to automate the process of identifying tweets manifesting evidence of depression symptoms. Although the dataset will not be openly available, the resulting, trained and tested natural language processing symptom classifiers will be openly available in the near future. These classifiers may be used to estimate and report the prevalence of other mental health disorders (eg, anxiety and eating disorders) by encoding shared symptoms and stressors leveraging similar language patterns from social media [33].

### Conclusions

We conducted a large-scale annotation study to investigate methods for effective data collection and understand how people tweet about depression on Twitter with the twin goals of (1) providing insights into how users express depressive symptoms on Twitter and (2) creating a dataset (ie, an annotated corpus of depression-related tweets) suitable for both training and testing natural language processing algorithms to automate the process of identifying tweets manifesting evidence of depression symptoms. We successfully developed an annotation scheme and an annotated corpus, the SAD corpus, consisting of 9300 tweets randomly selected from the Twitter API using depression-related keywords. Although the majority of tweets containing relevant keywords were nonindicative of depressive symptoms, several depressive symptoms and psychosocial stressor categories were observed including *depressed mood* and *fatigue or loss of energy*. In tweets containing two or more categories, we found correlations between some depressive symptoms and psychosocial stressor categories.

In summary, our analyses suggest that keyword queries alone might not be suitable for public health monitoring because the context can change the meaning of a keyword in a statement. However, postprocessing approaches could be useful for reducing the noise and improving the signal needed to detect depression symptoms using social media. We are actively investigating machine-learning based postprocessing as an approach to improve the precision of detecting depressive symptoms and psychosocial stressors [49,50].

## Abbreviations

API: Application Programming Interface
BRFSS: Behavioral Risk Factors Surveillance System
CLPsych: Computational Linguistics and Clinical Psychology
DSM-V: Diagnostic and Statistical Manual of Mental Disorders, Edition 5
DSM-IV: Diagnostic and Statistical Manual of Mental Disorders, Edition IV
HANDS: Harvard Department of Psychiatry National Depression Screening Day Scale
LIWC: Linguistic Inquiry and Word Count
NLP: natural language processing
NSDUH: National Survey on Drug Use and Health
PHQ-5: Patient Health Questionnaire
QIDS-SR: Quick Inventory of Depressive Symptomatology
SAD: Depressive Symptom and Psychosocial Stressors Acquired Depression
YRBSS: Youth Risk Behavior Surveillance System
